# MAROCOVID: Snapshot Monitoring of Knowledge and Perceptions of Safety Behaviors during the COVID-19 Outbreak in Morocco

**DOI:** 10.3390/ijerph18115745

**Published:** 2021-05-27

**Authors:** Imane Berni, Aziza Menouni, Younes Filali Zegzouti, Marie-Paule Kestemont, Lode Godderis, Samir El Jaafari

**Affiliations:** 1 Cluster of Competency “Health and Environment”, Moulay Ismail University, Meknes 50000, Morocco; y.filalizegzouti@fste.umi.ac.ma (Y.F.Z.); s.eljaafari@umi.ac.ma (S.E.J.); 2 Environment and Health Unit, Department of Public Health and Primary Care, Katholieke Universiteit Leuven, 3000 Leuven, Belgium; lode.godderis@kuleuven.be; 3 Institute for the Analysis of Change in Contemporary and Historical Societies, Université Catholique de Louvain, 1348 Louvain-la-Neuve, Belgium; marie-paule.kestemont@uclouvain.be; 4 IDEWE, External Service for Prevention and Protection at Work, 3001 Heverlee, Belgium

**Keywords:** COVID-19, knowledge, attitude, perceptions, safety behavior

## Abstract

To assess whether knowledge, attitude, and perceptions of the COVID-19 pandemic predicted changes in behaviors among the general Moroccan population, a cross-sectional online survey was conducted between 30 March and 20 April involving a total of 14,157 participants. The statistical analysis of the data included univariate and multivariate logistic regression analysis. Our results suggest that less than ten days after the Moroccan government announced “Health state of Emergency” response to the COVID-19 outbreak, public knowledge, attitude and responses to the pandemic were relatively high. More than half the respondents (63.2%) reported that they complied with more than five of nine recommended safety measures, including avoiding going out (93.2%), and frequent handwashing with soap and water (78.2%). Factors associated with an increased likelihood to adopt safety measures included perceptions that COVID-19 was a human health risk, the pandemic will continue for a long time, availability of clear information, and a lack of medicine. The largest predictor of safety behavior change was age; participants older than 55 were more likely to adopt recommended safety behaviors. Although knowledge and perception among the general public was reasonable, more encouragement from government via health education programs is needed to maintain appropriate behaviors.

## 1. Introduction

SARS-CoV-2 is the severe acute respiratory syndrome coronavirus 2 (COVID-19) that emerged in December 2019 in Wuhan, China [1] and was declared as a pandemic by the World Health Organization (WHO) on 11 March 2020 [2]. COVID-19 caused a global pandemic, with 78 million confirmed cases in more than 216 countries by 25 December 2020; there have also been 655,041 confirmed deaths across the globe, as reported by WHO [2]. Morocco saw an increase from 1 case on 2 March to 425,864 on 25 December and 7130 deaths [3].

In the frontline fight against COVID-19, vaccination is vital for achieving herd immunity [4]. Indeed, keeping mortality as low as possible was the highest priority for the health system and authorities worldwide. In turn, during the early stages of the spread of the pandemic strain of COVID-19, the Moroccan government implemented several mitigation measures to prevent the person-to-person spread of the disease. One of the most common interventions in the early weeks of the pandemic was a non-pharmaceutical measure including school or workplace closure, travel restrictions, case isolation, personal protection and hygiene, household quarantine, and social distancing, all of which are recommended by WHO [5]. These recommendations include frequent handwashing with soap and water or alcohol-based hand gel, covering mouth and nose with a disposal tissue when coughing or sneezing, avoiding touching eyes, nose and mouth [6], and using face masks. At the community level these measures may safeguard better physical and mental health during the COVID-19 pandemic [7].

Motivating the public to adopt such behaviors has been shown to be useful in containing the previous epidemic of infectious disease [8]. Likewise, the necessity of specific communication education for society to improve compliance with hygiene practices has proved to be paramount to prevent the spread of COVID-19 [9]. Participants consumed most of their COVID-19 information via “Internet, online newspapers, social networks” [10,11]. Thus, the training curriculum should include both theoretical approaches and contextual approaches to achieve efficient epidemic control [12]. However, while it is crucial to encourage the public to change their behavior in order to reducing the transmission of COVID-19, it can be difficult. Evidence shows that a perceived lack of confidence in the authorities, objectivity, and sincerity of the media in response to a public crisis could lead to district and fear [13]. However, helping people to change their behavior, protect themselves, and follow recommended measures means focusing on increasing their motivation by leveraging their knowledge/attitude [14], perceiving recommended measures characteristics, and being easily understood [15]. In addition to these factors, two other constructs were added. A greater feeling of threat from an outbreak will lead to greater uptake of safety measures [16]. Secondly, an individual is eager to adopt precautionary behaviors if he/she believes that the crisis will last a long time [16].

As we move into the delay phase of managing the COVID-19 pandemic (WHO), it becomes necessary to change people’s safety measures in relation to behavior across society as a method of flattening the pandemic peak. In this context, the Moroccan COVID-19 snapshot monitoring study (MAROCOVID) was initiated at the start of the COVID-19 outbreak, to assess whether knowledge, attitude and perceptions of the COVID-19 pandemic predicted changes in behavior among the general public. Furthermore, this study intends to support national authorities in managing the crisis and responding with effective and appropriate interventions, and policies that increase public health awareness about personal hygiene and safety measures.

## 2. Materials and Methods

### 2.1. Cross-Sectional Online Survey

This cross-sectional survey was conducted from 30 March to 20 April, ten days immediately after the lockdown of Morocco. The participants clicked the link on the platform and consented to respond voluntarily and anonymously to the survey. The eligibility criteria were as follows: (1) subjects aged 18 or above; (2) subjects who reported not having been infected with COVID-19. A de-duplication protocol was applied to identify multiple submissions to preserve data integrity, including cross-validation of the eligibility criteria and discrepancies in key data as well as checking for unusually fast completion time (<15 min) [17]. All research was conducted with generally accepted ethical principles and was previously approved by the Ethics Committee of Moulay Ismail University of Meknes (N: CERB-UMI 03/2020).

#### 2.1.1. Participants

A total of 15,008 participants clicked on the survey link, and 14,359 individuals commenced the survey, of whom 101 individuals refused to provide informed consent and 14,258 participants provided informed consent and submitted the questionnaires. After excluding 50 participants who did not complete all the questions and 51 respondents who were younger than 18 years, the final sample consisted of 14,157 participants.

#### 2.1.2. Behaviors

Participants were asked thirteen questions about recent behaviors in relation to the COVID-19 outbreak. Nine concerned the behaviors recommended by WHO [5] and the government [18], in relation to avoiding places or activities, practices of handwashing, mouth and nose protection. The four others were related to activities that had not been recommended—namely, following a healthy diet to boost the immune system, talking with a doctor about health issues related to COVID-19, wearing gloves when leaving home, and taking alternative supplements during the pandemic. Table 1 lists the full item wording. A 5-point Likert-scale (always, often, sometimes, rarely, never) was used to categorize the answers.

#### 2.1.3. Knowledge

A COVID-19 knowledge questionnaire had 16 questions (Table S1), four regarding COVID-19 nature and treatments, three concerning transmission routes, seven regarding main signs and symptoms, and two concerning incubation period. These questions were answered on a yes/no basis with an additional “I don’t know” option. A scoring system was applied to assess the level of knowledge of each subject: A correct answer was assigned 1 point and an incorrect/unknown answer was assigned 0 points. The total knowledge score ranged from 0 to 16.

#### 2.1.4. Attitude

Five questions measured attitudes toward COVID-19. Participants were asked about their belief that a specific action reduced the risk of caching COVID-19, with a possible response option being strongly agree (5), tend to agree (4), neither agree nor disagree (3), tend to disagree (2), and strongly disagree (1) (Table 2). A scoring system was applied using the Likert 5-point scale: 5 points were assigned to “strongly agree” and 1 point to “strongly disagree”. Thus, the total attitude score ranged from 6 to 25 points.

#### 2.1.5. Perceptions

Twenty-seven items were used to evaluate key themes of how people perceived the COVID-19 outbreak: Severity of COVID-19 (Sev) (three items), influence of the media (Med) (four items), control (three items), subjective norms (SN) (five items), timeline of the outbreak (Tim) (five items), confidence in the authorities (Conf) (five items), clear information (Inf) (one item), and lack of medications (one item) (Table S2). In asking people to present their opinion (disagreeing or agreeing) with the items offered, the present study employed a 5-point Likert-type scale, ranging from strongly agree (5) to strongly disagree (1). These items were adopted from the work of Rubin et al. [16].

Face and content validity of the questionnaire were examined to assure that the items could subjectively/theoretically cover the constructs [19]. For this purpose, a review panel consisting of two specialists at the Faculty of Sciences in Meknes (a professor of epidemiology and a professor of biological sciences) was formed to assess the research instrument. Their feedback was incorporated to enhance the readability, completeness, and clarity of the questions. The questionnaire was piloted on a small sample of participants from the general population prior to the study (n = 86). Data from this pilot sample were not used in the subsequent analysis. As a result, the clarity and appropriateness of the questions were evaluated, and the questionnaire was edited accordingly. Additionally, Cronbach’s alpha was employed to examine internal consistency reliabilities of the questions. As Table S2 shows, all construct values were either close to or above 0.70, showing adequate reliability of the questionnaire (ranging from 0.69 to 0.89).

#### 2.1.6. Personal Variables

Personal variables consisted of gender, age, marital status, educational level, employment status, any chronic disease diagnosed by a doctor, children in the household aged 0 to 5, and annual family income.

### 2.2. Statistical Analysis

Data analysis was performed using SPSS statistical software version 20.0 (Insight, Grimbergen, Belgium) and AMOS version 23.0 (Insight, Grimbergen, Belgium ). Frequencies, percentages, means, and standard deviations were used for the description of data. To determine the association between personal characteristics and safety behaviors (participants engaged in five of nine behaviors and one of four advised behaviors), a binary logistic regression analysis was performed, and the association between personal variables and behaviors are presented as odds ratios (ORs) and 95% Cls. Two sets of binary logistic regressions were used to assess the univariate associations between perception variables and primary outcomes, and also the multivariate associations adjusting for significant personal variables including age, gender, marital status, education level, employment status, chronic disease, presence of children, and monthly family income. Pearson’s correlation test was used to find the correlation between knowledge, attitude, and following more than five recommended behaviors.

## 3. Results

This cross-sectional survey was conducted ten days immediately after the lockdown of Morocco. Because it was not feasible to carry out a community-based national sampling survey during this particular period, we decided to collect data online. Relying on social networks and mass mailing, an online questionnaire was spread via Facebook, WhatsApp, Instagram, Twitter, and LinkedIn using sponsored social network advertisements. We used a convenience sampling method. The members clicked the link on the platform and responded to the survey until the convenience sample covered all 12 regions in Morocco. A total of 14,157 respondents participated in the survey. Among the survey completers, 63.7% were women, 73.7% aged 25 to 54, 86.2% held a Bachelor degree or above; other demographic characteristics are shown in Table 3.

### 3.1. Behavior Outcomes, Knowledge, and Attitude toward COVID-19 Pandemic

Table 1 shows the behavioral changes recorded in response to the COVID-19 pandemic. The surveyed participants informed thirteen possible safety behaviors (SBs) in relation to COVID-19. Overall, 8946 of the respondents (63.2%) adopted more than five of the recommended SBs. Avoiding going out was the most common SB practice, followed by Increasing the amount I clean or disinfect things that I might touch. Whereas, 3933 (27.8%) reported engaging in one or other form of the four behaviors classified as advised. Regarding knowledge assessment, 12,087 people (85.2%) were aware that the disease is contagious, and a large number have good knowledge about the COVID-19 transmission route and principal symptoms (fever, cough, breathing difficulty, and sore throat) (Table S1). Table 2 lists participants’ attitudes towards the COVID-19 outbreak.

### 3.2. Personal Variables Associated with Safety Behaviors

Binary logistic regression analysis showed that, after controlling for confounders, being a woman, married, a parent, and having a chronic disease meant that you were significantly more likely to follow recommended behaviors (e.g., among women, adopting more than five recommended behaviors: OR, 1.8; 95% Cl, 1.7–1.9; *p* < 0.001). Compared with working full- or part-time, not working was associated with following recommended behaviors (OR, 1.5; 95% Cl, 1.4–1.6; *p* < 0.001). Participants with a monthly household income of 8000 MAD to 12,000 MAD (approximately 800 to 1200 EUR) or with higher education qualifications were significantly more likely to adopt recommended behaviors (OR, 1.2; 95% Cl, 1.1.–1.3; *p* < 0.001, OR, 0.8; 95% Cl, 0.7–0.9; *p* < 0.001) respectively. The strongest predictor for behavior change was being in the age group 55 to 64; these participants were significantly more likely than other groups to adopt advised safety behaviors (OR, 4.5; 95% Cl, 0.9–21.6; *p* < 0.05) (Table 3).

### 3.3. Attitude towards Specific Behaviors as a Predictor of Behavioral Change

Binary logistic regressions showed significant univariate associations between attitude towards avoiding going out and adopting >5 recommended behaviors (OR, 0.92; 95% Cl, 0.86–0.98), attitude towards using gloves and face mask when out in public (OR, 1.04; 95% Cl, 0.99–1.08), attitude concerning washing hands regularly with soap and water (OR, 0.98; 95% Cl, 0.956–1.02), attitude towards avoiding shopping at supermarkets and crowded places (OR, 1.002; 95% Cl, 0.98–1.01), and attitude towards following a healthy diet could boost my immune system (OR, 0.97; 95% Cl, 0.95–1.00) (Table S3).

### 3.4. Correlation between Knowledge, Attitude and Safety Behaviors

A linear relationship was found between knowledge and attitude, knowledge and behavior, and knowledge and behavior using Pearson’s coefficient (Table S4). According to the findings, significant associations were found between knowledge and behaviors (0.674) and attitude and behaviors (0.443), suggesting that an increase in knowledge and positive attitude would lead to an increase in behavioral changes.

### 3.5. Correlation between Perceptions and Safety Behaviors

Table 4 indicates the mean scores for the perception factors and the univariate and multivariate correlations between perceptions and safety behaviors. Adjusting for all personal variables in Table 4, all perceptions were associated with following five or more recommended behaviors. The largest effects were for respondents who perceive that COVID-19 is severe and there is no treatment; they were significantly more likely to adopt both recommended and advised behaviors (e.g., severity of COVID-19: OR, 1.6; 95% Cl, 1.5–1.7; *p* < 0.001 for carrying out more than five recommended behaviors: OR, 1.2; 95% Cl, 1.1–1.3; *p* < 0.001 for carrying out more than one advised behavior). Even perception relating to severity of the illness, influence of the media, subjective norms, timeline of COVID-19, confidence in the authorities, good information and lack of treatment also demonstrated significant associations with undertaking one or more advised behaviors.

## 4. Discussion

Over the past few months the spread of COVID-19 has had a huge impact on our behaviors. All human beings are now facing great challenges due to the pandemic. This study assessed public perceptions, knowledge, attitude, and behavioral change related to the COVID-19 pandemic in Morocco. Our results suggest that less than ten days after the Moroccan government announced a “State of Health Emergency” response to the COVID-19 outbreak, public knowledge, attitude and responses to the pandemic were relatively high. More than half of the respondents reported complying with more than five of the ten recommended safety behaviors, including avoiding going out, and frequent handwashing with soap and water for more than 20 s. The practice of these protective behaviors has played an important role in controlling the spread of COVID-19 [20,21]. This finding may support the postulation that personal precautionary measures could offer psychological benefits, such as feeling less vulnerable to infection [20].

Helping the public to undertake safety measures during the COVID-19 outbreak is a challenging task. If a community is to change its responses rapidly, the influence of the media and the availability of clear information are critical factors. Our results showed that right from the initial phase of the pandemic, the majority of the general public closely followed the information about the pandemic, via multiple kinds of media. Therefore, the public were well supplied with the most updated information, which may explain the high level of knowledge and the positive attitude. A previous study has demonstrated that misinformation and false reports about COVID-19 may confuse people and harm people’s mental health [22]. Thus, satisfaction with health information could be treated as a protective factor for mental health during the COVID-19 pandemic. Knowledge is a strong determinant of attitude, shaped by acquired information and experiences. It is therefore widely accepted that knowledge drives attitudes towards behavior [23]. People’s knowledge of the COVID-19 risk was positively related to attitudes toward the adoption of safety measures. In turn, insufficient knowledge of the COVID-19 pandemic implied a higher risk of exposure [23]. Thus, it is crucial to provide people with the right knowledge during such situations for the effective prevention of disease spread [14]. Therefore, improving people’s knowledge about COVID-19 should be the first goal in minimizing exposure to the pandemic. Our findings are comparable to several epidemic/pandemic studies. The largest previous study examining associations between knowledge, attitudes and practices towards COVID-19 used an online survey with 6910 Chinese residents [24]. The study reported that the majority were knowledgeable about the COVID-19 pandemic, and had an optimistic attitude towards COVID-19. Another study reported that the Indian population have an adequate knowledge about preventive aspects, and the attitude towards COVID-19 showed people’s willingness to follow government guidelines on quarantine and social distancing [25]. Nevertheless, in a study conducted in Trinidad and Tobago in 2016, following the H1N1 outbreak, it was observed that a significant proportion of the general public was unaware of the seriousness of the epidemic and related measures [14]. A similar study, evaluating the knowledge, attitude and perception regarding the Ebola virus infection among secondary school children in Nigeria, found that most of the participants had poor knowledge and had a negative attitude toward the epidemic [26]. In this regard, public perceptions and attitudes toward preventive measures are vital to ensure the success of a national response in combating COVID-19. Similarly, previous studies have found that the success of government response strategies relied heavily on the public’s perceptions and attitude toward the risk of SARS epidemic and the importance of control measures [27]. Another research study showed a high level of agreement among the general population about the importance and necessity of national response measures to combat the COVID-19 pandemic [28]. Therefore, in order to maintain appropriate attitudes, governments may need to use realistic portrayals (community stories) and role modelling by influential actors in social networks. Observing competent role models perform actions that result in success conveys information to observers about the sequence of actions needed for success [29]. Motivation may be helped by creating media campaigns that foster awareness of the recommended behaviors and encourage people to share their strategies for complying with self-isolation and working from home. The SBs of the MAROCOVID study were strongly influenced by numerous personal variables. The findings of independent associations of gender, age, marital status, and family income is in line with the existing literature [24,30,31]. Findings from this study show that the respondents who were more likely to adopt high safety behavior were women. Previous research has reported that women are more likely to perceive themselves as vulnerable and hence to adopt protective measures [32]. However, a study in Australia showed that male respondents were more likely to engage in preventive measures [33]. Such gender role divisions are more sensitive to pandemic issues. Second, as age increases, people are more likely to adopt recommended and advised protective behaviors. This finding is in agreement with those of Cvetković et al. [34] and Kim and Kim [35]. This study finding could be due to the fact that older people feel more susceptible to infection with COVID-19. Furthermore, a married status was a protective factor due to the values of family support among Moroccans. Married respondents were more likely to adopt recommended safety behaviors. This gap could be explained by the traditional role of the family in Morocco; family support has a greater influence on reducing the risk of adverse mental health.

In this study, respondents with higher incomes were significantly more likely to follow recommended behaviors. Economic affordability can help to minimize external risk by giving people access to resources to protect against the dangers of COVID-19. In Morocco, the economic situation caused by the COVID-19 pandemic has led to decreased incomes [36]. Such economic poverty undermines the successful prevention of COVID-19. Similar results were found in other countries [31,37,38]. However, conflicting results have been reported by some researchers who found that income does not have a statistically significant effect [34].

Furthermore, our results also correspond to a study conducted in the UK during a swine flu epidemic that reported a greater behavioral change among parents of young children and participants with a chronic disease [16]. Being parents of children aged <5 years was one of the factors affecting safety behaviors during the COVID-19 pandemic. This finding is interpreted to mean that individuals are engaged in more safety practices when family members are vulnerable to COVID-19.

As in other studies, enhanced safety measures were seen among the participants with higher education levels. A study from Korea showed that respondents with a college education or higher took more comprehensive precautionary measures relative to individuals with a lower education [35]. Duan et al. also suggested that a longer education period is associated with more engagement in government-recommended behavior [39]. In contrast to the above studies, Cvetković et al. [34] empirically reported that education level does not affect precautionary measures. Thus, educational programs for this population could be an effective option to remedy this issue.

The MAROCOVID study makes an important contribution by addressing key limitations in the previous literature related to COVID-19 outbreaks, and by showing that the perception that the pandemic represents numerous threats to human health and will continue for a long time increases the safety behavior adherence. This fatalistic conclusion was observed in some people during the swine flu epidemic in the UK [16]. This implies that people with a high perception of the severity of the COVID-19 pandemic consequences will be more likely to change their behavior. From a psychological perspective, individuals take action in the area of health if they believe that the harm can be serious [40]. The severity of the health risk is a key feature in shaping people’s behavior and convincing people to adopt more safety measures. In fact, on the day our survey questionnaire was finished, there were more than 2 million confirmed cases of COVID-19 and about 157,955 deaths worldwide [2]; this health threat is accepted as an indication that it is essential for future outbreaks to alter public behavior towards safety measures. Musa Ibrahim, 2015, reported that severity includes beliefs about the disease itself as well as the broader impacts of the disease on an individual’s social role [41]. This, in turn, may lead to bigger changes in behavior to avoid these impacts. Transmission dynamic models of the COVID-19 outbreak show that containment played a major role in limiting the spread of infection in Morocco [42]. Taking this into account, the promotion of extension services and public awareness campaigns to sensitize the public about the persistent risk of COVID-19 even after the end of the containment can also be regarded as a key policy option.

Most of our samples agreed that there is no medicine to treat people with COVID-19 (mean score 4.5 of 5). This perception was correlated with an increased probability to follow recommended and advised behaviors. Our estimates are generally higher than in the UK population study [16,43], probably because of the high threat presented by the COVID-19 pandemic compared to other infectious diseases. Indeed, less confidence in the Moroccan government being able to win the battle against COVID-19 was also associated with a lower likelihood of following safety measures. The importance of fostering greater trust between government and the general public has been suggested before [30], and more efforts should be made in this area.

Despite the importance of SN as a significant predictor in changing behavior during the COVID-19 outbreak, the result of the study showed that this component had a weak correlation with safety behaviors compared to other factors. This result is disputable since changing behaviors is not known as a strong SN among Moroccan people, as a result of which they are under no social pressure to change their behavior during the quarantine period. This is particularly true of the legal pressure coming from the government. During the quarantine period in Morocco, the government introduced some regulations regarding safety measures to protect people from COVID-19, including the obligation for people to wear facemasks when going out; anybody refusing to wear a facemask was liable to the sanctions stipulated in article 4 of the legislative decree 2.20.292. Despite this, there are some people who are still not familiar with these regulations and consequently often ignore them when going out. It is clear from this that governmental supports play an essential role in the promotion of measures required for peoples’ occupational safety and health.

The findings of this research indicate several implications that can be helpful in future COVID-19 and other pandemics in Morocco. First, according to previous research, there may be other structural variables affecting people’s risk perceptions of COVID-19. People’s perceived susceptibility to COVID-19 plays a key role in determining whether they take protective measures [35]. An increase in perceived susceptibility to a particular health problem would cause them to engage in behaviors to reduce their risk of developing the health problem [44]. Thus, people’s perceived susceptibility to COVID-19 should be measured regularly to ensure that people engage in effective behaviors. Second, given that COVID-19 is still not under control worldwide, policymakers are hoping to contain this pandemic through vaccination. Around 50 candidate vaccines are currently under clinical evaluation [45]. However, the effectiveness of a vaccination program is limited if people refuse to take part in it [46]. Perceived COVID-19 susceptibility, low potential risk of vaccine harm, and solidarity as the main drivers for COVID-19 vaccination should be examined.

Our study has several limitations: first, when compared to the current population statistics in Morocco [47], our sample was obviously over-representative of women, well-educated people, and people working full- or part-time, who were also likely to adopt suggested safety behaviors. This should not be generalized to the whole population. Secondly, the MAROCOVID study was carried out in the early stages of the COVID-19 outbreak in Morocco, when the infection rate was gradually increasing. The daily infection rate of COVID-19 was more than 200 cases per day [2] during baseline data collection (30 March to 20 April 2020), and it further declined to about 40 cases per day during the 1-month follow-up. This could have affected the perceived importance of safety measure adherence. Further follow-up could be useful to monitor changes in behaviors and practices. Third, the current study focused on assessing perceptions and attitudes toward the main safety measures communicated to the public during the COVID-19 pandemic; while, this study is not based on theories and models. However, in analyzing the impact of proactive behaviors, the theory of planned behaviors, which emphasizes the determinants of behavioral actions [35]; a risk communication model that explores the social spread of risk [48] can be used as a model for identifying a resource factor that influences individuals’ preventive actions in response to COVID-19 for further studies.

## 5. Conclusions

To the best of our knowledge, this is the first study that has quantified the impact of knowledge, attitudes and perceptions toward COVID-19 among Moroccan residents. The MAROCOVID study showed that for the COVID-19 outbreak to be controlled, both government, media, and community efforts need to be considered. The increase in knowledge and attitude greatly contributes to the control of the pandemic in Morocco. Therefore, the surveillance system should not only include the number of infections and deaths, but should also monitor changes in perceptions and behaviors by carrying out systematic surveys, such as the one in this study. These surveys should be conducted during the initial onset of the pandemic and in real time, and should include a feedback mechanism to policymakers and the general public so that areas of concern can be addressed and related perception and behaviors can be reinforced. Lastly, although the quarantine program was well supported, the community was dissatisfied with the government’s responses and doubtful of its ability to control the pandemic. We suggest that public health authorities should attempt a “person-centered” approach rather than a “disease-centered” approach when prioritizing policies and communication efforts to accommodate all the needs.

As the effect of COVID-19 is unclear at present, encouraging the community to maintain appropriate behaviors could be fundamental. In order to be engaged, the public needs to feel that they are a valued member of a team and that their efforts are important and key to the overall response. It is also essential that government ensures that resources, legislation and support measures are in place in order to facilitate community engagement in community mitigation strategies.

Finally, the MAROCOVID study believes that this preliminary report includes useful information that can not only guide communication and mitigation strategies during COVID-19 but can also inform future pandemic preparedness planning and identifying ways of encouraging behavior change during a potential second wave of the COVID-19 pandemic.

## Figures and Tables

**Table 1:** Safety behavioral responses to the COVID-19 pandemic.

Interview Question	N (%) of Positive Responses
Over the past ten days. I have … to prevent infection from COVID-19	
- Avoided going out *	13,201 (93.2)
- Washed hands with soap and water frequently and for more than 20 s *	11,073 (78.2)
- Worn a face mask when leaving home *	10,614 (75)
- Avoided touching eyes, nose and mouth with unwashed hands *	8335 (58.9)
- Increased the amount I clean or disinfect things that I might touch *	12,677 (89.5)
- Used disinfectant (alcohol) to clean hands *	8325 (58.8)
- Reduced my use of public transport *	7021 (49.6)
- Reduced the number of times I go into shops *	12,094 (85.4)
- Maintained at least a 1-m distance between myself and others *	7909 (55.9)
- Followed a healthy diet to boost my immune system +	6374 (44.8)
- Talked with a doctor about health issues related to COVID-19 +	4154 (29.3)
- Taken alternative supplements during the pandemic +	3987 (28.2)
- Worn gloves when leaving home +	5776 (40.8)
Participants engaged in more than 5 of the 10 recommended behaviors	8946 (63.2)
Participants engaged in more than 1 of the 3 advised behaviors	3933 (27.8)

* Behavioral changes classified as recommended; + Behavioral changes classified as advised.

**Table 2:** Attitude of participants towards COVID-19 outbreak.

Question	Strongly Agree	Tend to Agree	Neither Agree nor Disagree	Tend to Disagree	Strongly Disagree
I think that ... reduces my risk of catching COVID-19					
-Avoiding going out	11,952 (84.4)	1692 (12.0)	324 (2.3)	135 (1.0)	54 (0.4)
-Using gloves and face mask when out in public	7020 (49.6)	4887 (34.5)	1638 (11.6)	513 (3.6)	99 (0.7)
-Washing hands regularly with soap and water	9936 (70.2)	2331 (16.5)	612 (4.3)	891 (6.3)	387 (2.7)
-Avoiding shopping at supermarkets and crowded places	9036 (63.8)	2178 (15.4)	801 (5.7)	1584 (11.2)	558 (3.9)
-Following a healthy diet could boost my immune system	4077 (28.8)	1224 (8.6)	3772 (25.9)	3870 (27.3)	1314 (9.3)

**Table 3:** Association between personal variables and safety behavior during COVID-19 identified by binary logistic regression analysis.

Variable and Variable Levels	No (%) of Participants	No (%) Engaged in Recommended Behaviors	Odds Ratio (95% Cl)	*p*-Value	No (%) Engaged in Advised Behaviors	Odds Ratio (95% Cl)	*p*-Value
**Gender**							
Women	9018 (63.7)	6165 (60.3)	1.8 (1.7–1.9)	0.000	1629 (18.1)	1.4 (1.3–1.5)	0.000
Men	5139 (36.3)	2781 (54.1)	Reference	NA	2232 (43.4)	Reference	NA
**Age group**							
18–24	2979 (21)	1647 (55.3)	2.3 (1.6–3.2)	0.000	783 (26.3)	2.5 (0.6–11.5)	0.012
25–34	6183 (43.7)	4032 (79.8)	3.4 (2.5–4.8)	0.000	1809 (29.3)	3.1 (0.7–13.9)	0.012
35–54	4248 (30)	2808 (66.1)	3.6 (2.6–5)	0.000	1053 (24.8)	2.5 (0.5–11.4)	0.21
55–64	594 (4.2)	405 (68.2)	3.9 (2.7–5.7)	0.000	207 (34.8)	4.5 (0.9–21.6)	0.043
>64	153 (1.1)	54 (35.2)	Reference	NA	9 (5.9)	Reference	NA
**Marital status**							
Married	7119 (50.3)	4716 (66.2)	1.3 (1.1–1.6)	0.012	1728 (24.3)	0.8 (0.6–1.0)	0.007
Others	7038 (49.7)	4230 (60.1)	Reference	NA	2133 (50.4)	Reference	NA
**Educational level**							
None	198 (1.4)	18 (9)	0.04 (0.02–0.06)	0.000	54 (27.3)	0.9 (0.6–1.2)	0.42
Primary school	693 (4.9)	72 (10.4)	0.04 (0.03–0.06)	0.000	153 (22.1)	0.6 (0.5–0.8)	0.000
Secondary school	2097 (14.8)	981 (46.7)	0.3 (0.2–0.4)	0.000	549 (26.2)	0.6 (0.5–0.7)	0.000
Bachelor university	5067 (35.8)	3429 (67.7)	0.8 (0.7–0.9)	0.000	1368 (27.0)	0.7 (0.6–0.8)	0.001
Master & doctorate	6102 (43.1)	4446 (72.9)	Reference	NA	1827 (27.2)	Reference	NA
**Working position**							
Not working	5499 (38.8)	3150 (57.3)	1.5 (1.4–1.6)	0.000	1503 (27.3)	0.9 (0.7–1.1)	0.001
Working full- or part-time	8658 (61.2)	5796 (66.9)	Reference	NA	2358 (27.2)	Reference	NA
**Chronic disease**							
Present	4275 (30.2)	3069 (71.8)	1.7 (1.6–1.9)	0.000	1053 (24.6)	1.3 (1–1.6)	0.014
None	9882 (69.8)	5877 (59.5)	Reference	NA	2808 (28.4)	Reference	NA
**Children in household**							
Aged <5	7956 (56.2)	5679 (71.3)	2.2 (1.8–2.7)	0.000	2511 (31.5)	1.5 (1.2–1.9)	0.001
Aged >5 or no children	6201 (43.8)	3267 (52.6)	Reference	NA	1422 (22.9)		NA
**Monthly family income**							
<2000 dh	4491 (31.7)	2601 (57.9)	0.8 (0.7–0.9)	0	1188 (26.5)	0.7 (0.5–1.0)	0.07
2000–4000	720 (5.1)	450 (62.5)	1.0 (0.8–1.1)	0.601	198 (27.5)	0.8 (0.4–1.3)	0.45
4000–8000 dh	1719 (12.1)	1161 (67.5)	1.1 (1.0–1.3)	0.004	387 (22.5)	0.6 (0.4–0.9)	0.02
8000–12,000 dhs	3429 (24.2)	2322 (67.7)	1.2 (1.1–1.3)	0	954 (27.8)	0.8 (0.6–1.1)	0.01
>12000	3798 (26.8)	2412 (63.5)	Reference	NA	1206 (31.8)	Reference	NA

**Table 4:** Correlation between perception factors and behavior during COVID-19 pandemic.

Factors	Mean (SD) Score **	Association with Carrying Out >5 Recommended Behaviors		Association with Carrying Out >1 Advised Behaviors	
		Odds Ratio (95%Cl)	Adjusted Odds Ratio (95%Cl) +	Odds Ratio (95%Cl)	Adjusted Odds Ratio (95%Cl) +
Severity of COVID-19	4.2 (0.84)	1.6 (1.5–1.7)	1.5 (1.4–1.6)	1.2 (1.1–1.3)	1.1 (1.0–1.2)
Influence of media	3.2 (0.92)	1.3 (1.2–1.3)	1.2 (1.2–1.3)	1.0 (1.0–1.1)	0.9 (0.9–1.1) *
Control	4.3 (0.87)	1.3 (1.1–1.3)	1.1 (1.1–1.2)	1.1 (1.0–1.2)	1.1 (1.0–1.1) *
Subjective norms	3.8 (1.04)	1.2 (1.1–1.3)	1.1 (1.1–1.2)	1.0 (1.0–1.1) *	0.9 (0.9–1.1) *
Timeline of COVID-19	3.8 (0.72)	1.5 (1.4–1.6)	1.4 (1.3–1.6)	1.3 (1.2–1.4)	1.2 (1.2–1.4)
Confidence in the authorities	3.1 (1.3)	1.2 (1.1–1.3)	1.2 (1.1–1.3)	0.9 (0.8–1.0)	0.9 (0.9–1.0)
Lack of medications ++	4.5 (0.74)	1.6 (1.5–1.7)	1.6 (1.5–1.7)	1.1 (1.0–1.2)	1.1 (1.0–1.1) *
Clear information ++	4.0 (0.90)	1.3 (1.2–1.4)	1.3 (1.2–1.4)	1.1 (1.0–1.1) *	1.0 (1.0–1.1) *

* significant at *p* < 0.05; ** higher score indicates greater agreement; + adjusting for personal variables (age, gender, marital status, education level, employment status, chronic disease, presence of children, family income); ++ factors represented by single item that authors thought best illustrated underlying concept.

## Supplementary Materials

The following are available online at https://www.mdpi.com/article/10.3390/ijerph18115745/s1, Table S1: Knowledge about COVID-19 among Moroccan public, Table S2: Descriptive statistics of key variables toward COVID-19 (n = 14,157), Table S3: Correlation between knowledge, attitude and behavior, Table S4: Correlation between knowledge, attitude and behavior.
