# Supplementary material for: MAROCOVID: Snapshot Monitoring of Knowledge and Perceptions of Safety Behaviors during the COVID-19 Outbreak in Morocco
Source: Int J Environ Res Public Health. 2021 May 27;18(11):5745. doi: 10.3390/ijerph18115745 (PMC8198599; doi:10.3390/ijerph18115745)
Supplement: Supplementary file 1 [file ijerph-18-05745-s001.zip › ijerph-1197678-SI.pdf]

MAROCOVID: Snapshot monitoring of knowledge and perceptions of safety behaviors during the COVID-19 outbreak in Morocco

Imane Berni [1,*,†], Aziza Menouni [1,2,*,†], Younes Filali Zegzouti [1], Marie-Paule Kestemont [3], Lode Godderis [2,4] and Samir El Jaafari [1]

[1] Cluster of Competency "Health and Environment", Moulay Ismail University, Meknes 50000, Morocco; y.filalizegzouti@fste.umi.ac.ma (Y.F.Z.); s.eljaafari@umi.ac.ma (S.E.J.)
[2] Environment and Health Unit, Department of Public Health and Primary Care, Katholieke Universiteit Leuven, 3000 Leuven, Belgium; lode.godderis@kuleuven.be
[3] Institute for the Analysis of Change in Contemporary and Historical Societies, Université Catholique de Louvain, 1348 Louvain-la-Neuve, Belgium; marie-paule.kestemont@uclouvain.be
[4] IDEWE, External Service for Prevention and Protection at Work, 3001 Heverlee, Belgium
* Correspondence: imane.berni@gmail.com (I.B.); aziza.menouni@kuleuven.be (A.M.)
† These authors contributed equally to this work and are co-first authors.

Table S1: Knowledge about COVID-19 among Moroccan public.

Question	Yes	No	I do not no
Is COVID-19 pandemic contagious?	12087 (85,2)	207 (1,5)	1863 (13,2)
Is COVID-19 pandemic caused by a virus?	7110 (50,2)	1197 (8,5)	5850 (41,3)
Is there a vaccine against COVID-19?	7740(54,7)	1701 (12)	4716 (33,3)
Is there an effective treatment for people who have COVID-19	2025 (14,3)	7947 (56,1)	4185 (29,6)
Possible to contrast COVID-19 by:			
- Being close contrast with someone with COVID-19	13644 (96,4)	135 (1)	378 (2,7)
-Touching objects or surfaces that have been in contact with someone who has COVID-19	13536 (95,6)	216 (1,5)	405 (2,9)
Shaking hand with someone who is an active case of COVID-19	13545 (95,7)	261 (1,8)	351 (2,5)
Symptoms of the COVID-19 are:			
-Fever	13518 (95,5)	198 (1,4)	441 (3,1)
-Cough	13464 (95,1)	216 (1,5)	477 (3,4)
- Breathing difficulty	13473 (95,2)	342 (2,4)	342 (2,4)
- Sore throat	12051 (85,1)	531 (3,8)	1557 (11,1)
-Diarrhea	7641 (54)	3096 (21,9)	3420 (24)
-Lack of appetite / Headaches	6939 (49)	2475 (17,5)	2743 (33,5)
-Loss of smell / Fatigue	9774 (69)	1656 (11,7)	2727 (19,3)
14 days is the incubation period for COVID-19	4500 (31,8)	2448 (17,3)	7209 (50,9)
COVID-19 can affect people more than one in life	4968 (35,1)	3015 (21,3)	6174 (43,6)

Table S2. Descriptive statistics of key variables toward COVID-19 (n=14 157)

Factors	Variable description	Mean (SD)
**Severity of COVID-19**		
	I think that COVID-19 put several threats to human health	4,44 (0,88)
	I believe that if I catch COVID-19 it will have a serious consequence for my life	4,24 (0,86)
	I believe that COVID-19 can lead to death of my loves one if they get infected	4,02 (0,80)
**Cronbach's α**		0,76
**Influence of Media**		
	I think media platforms are fulfilling their job in raising awareness	4,1 (1,01)
	I think that the media have over-exaggerated the risks of catching COVID-19	3,42 (1,0)
	I follow the information about COVID-19 from Television and radio	2,48 (0,9)
	I follow the information about COVID-19 from Internet and social media (Facebook, Twitter, …)	3,1 (0,8)
**Cronbach's α**		0,69
**Control**		
	I may decrease my risk to catch COVID-19	4,34 (0,77)
	My behavior can prevent transmission	4,02 (0,96)
	I know how to protect myself and my beloved one	4,38 (0,9)
**Cronbach's α**		0,87
**Subjective norms**		
	The way that others think about COVID-19 is important to me	4,00 (0,99)
	My family discourages me to go out in this period	4,38 (0,96)
	My close friends confirm that COVID-19 is a serious disease	3,68 (1,01)
	I feel like under social pressure to wash my hand many times	3,68 (1,06)
	The persons I most respect, wear mask while going out	3,53 (1,22)
**Cronbach's α**		0,78
**Timeline of COVID-19**		
	In my opinion, COVID-19 is going to continue for a long time	4,74 (0,67)
	In my opinion, COVID-19 will be successfully controlled	1,8 (0,33)
	In my opinion, confirmed cases and mortality caused by COVID-19 will increase	4,26 (0,89)
	In my opinion, the lockdown will still until July	4,41 (1,05)

	In my opinion, Airspace will remain closed for six months at least	4,23 (0,71)
**Cronbach's α**		0,86
**Confidence in the authorities**		
	I think the authorities act in the interest of the public in dealing with COVID-19	3,89 (1,36)
	I think politicians usually tell us the true number of confirmed and death cases	3,94 (1,27)
	I think government closely monitor all citizens during COVID-19	3,92 (1,28)
	I think government of Morocco is handling the COVID-19 health and socioeconomic crisis very well	2,04 (1,23)
	I'm confident that Morocco can win the battle against the COVID-19 pandemic	2,11 (1,16)
**Cronbach's α**		0,89
**Lack of medications**	Nothing can be done to treat people with COVID-19	4,5 (0,74)
**Clear information**	The information I have heard about COVID-19 has been obvious	4,0 (0,9)

Table S3. Correlation between attitudes and behavior during COVID-19 pandemic

Factors	Association with carrying out> 5 recommended behaviours	Association with carrying out> 1 advised behaviours
	Odds ratio (95%Cl)	Odds ratio (95%Cl)
I think that, ….. reduce my risk of catching Covid-19 pandemic		
Avoiding going out	0,92 (0,86-0,98)	0,85 (0,84-0,95)
Using gloves and face mask when outing public	1,04 (0,99-1,08)	0,99 (0,95-1,04)
Washing hand regularly with soap and water	0,98 (0,95-1,02)	0,97 (0,93-1,01)
Avoiding shopping at supermarket and crowded place	1,00 (0,98-1,01)	0,95 (0,92-0,98)
Following a healthy diet could boost my immune system	0,97 (0,95-1,00)	0,92 (0,91-0,99)

Table S4. Correlation between knowledge, attitude and behavior

Relationship between		Coefficient of correlation
Knowledge score	Attitude score	0.492
Knowledge score	Behavior	0.674
Behavior	Attitude score	0.443
